# Supplementary material for: Laser printed nano-gratings: orientation and period peculiarities
Source: Sci Rep. 2017 Jan 9;7:39989. doi: 10.1038/srep39989 (PMC5220325; doi:10.1038/srep39989)
Supplement: Supplementary Information [file srep39989-s1.doc]

**Supplementary information:**

**Laser printed nano-gratings: orientation and period peculiarities**

Valdemar Stankevič, ^1, *^ Gediminas Račiukaitis, ^1^ Francesca Bragheri, ^2^ Xuewen Wang, ^3^ Eugene G. Gamaly, ^4^ Roberto Osellame, ^2^ and Saulius Juodkazis^3^

^1^Center for Physical Sciences and Technology, Savanoriu Ave. 231, Vilnius LT-02300, Lithuania

^2^Istituto di Fotonica e Nanotecnologie - CNR, P.za Leonardo da Vinci 32, I-20133 Milano, Italy

^3^Center for Micro-Photonics, Faculty of Science, Engineering and Technology, Swinburne University of Technology, John St., Hawthorn, Melbourne VIC 3122, Australia

^4^Laser Physics Centre, Research School of Physics & Engineering,

The Australian National University, Canberra, Australia

(Dated: August 1, 2016)

**Temporal and spatial chirp**

Dependence of the nanograting tilt on the temporal pulse chirp, $\gamma$, was measured by changing the pulse compressor grating position, hence, varying the second order dispersion of the fs-laser pulse. The compressor position for the shortest pulse duration corresponded to the chirp-free or Fourier transform limited bandwidth pulses with the chirp parameter$\gamma=0 \text{fs}^{-2}$. The pulse duration was measured by a frequency-resolved optical gating (FROG) algorithm using a second harmonic auto-correlator (Ekspla, Ltd).

The pulse duration at full width half maximum (FWHM) was determined using the FROG numerical retrieval. Temporal dependence of the Gaussian pulse was considered in the form: $E(t)=E_{0}e^{-2\ln2(t/\tau_{p})^{2}}\cos(\omega t+\gamma t^{2})$, here $\omega$ is the cyclic frequency of light at the central wavelength, *t* is the time, $\tau_{p}$is pulse duration at FWHM, $\gamma\text{(fs}^{-2}\text{)}$ is the chirp, and $E_{0}\equiv\sqrt{2I_{0}/(c\varepsilon_{0}n)}$ is the E-field amplitude. The spatial chirp, i.e. the pulse front tilt, was measured using a commercial single-shot autocorrelator (TiPA, Light Conversion Ltd.). The measurements were carried out directly on the target by mounting TiPA device instead of the sample without a microscope objective. In this, way any tilt induced on the optical path was taken into account. The pulse compressor design in the used lasers can affect the pulse front tilt at the laser output (along the x-axis), i.e., when scanned at $\phi=\pi/2$ direction, there could be the difference in the pulse arrival time on the target at the outmost left and right parts of the focal spot, a “snow plow” effect (inset in Fig. S1(c)). The temporal and spatial chirps were measured for this $\phi=\pi/2$ condition. It is noteworthy, such measurement characterises laser pulses of the fundamental wavelength of 1030 nm and not directly of the second harmonics at 515 nm that was also used in the nanograting formation; pulse duration at SHG is estimated from a $I^{2}$ envelope of the Gaussian pulse. The second harmonics pulses are usually preferable for laser structuring since the excitation of plasma by a nanosecond pedestal that presents for the pulses at fundamental frequency was avoided^1^ (peak the intensity of the pedestal is only$10^{-3}$, however, the entire energy can be substantial due to long ~ 20 ns duration). Figure S1 summarises experimental observations of the chirp measurements.

The temporal chirp was tuned by the pulse compressor stage position (Fig. S1(a, b)). Around the shortest pulse $\tau_{p}=260 \text{fs}$ setting of the compressor stage, there was a sign change of the nanograting tilt angle from negative values $\Psi<0$ (at$\gamma>0$) to the positive $\Psi>0$ (at$\gamma<0$), however, the overall effect was not strong. Experiments showed that no tilt can be expected $\Psi\cong0$ for the shortest pulse ($\gamma=0$). For strongly negatively chirped pulses, a tilt of nanogratings returns to negative values $\Psi<0$ (Fig. S1(a)).The front tilt measurements were carried out with a 2-mm-diameter beam at the target location using Pharos laser. The pulse front tilt at the compressor settings around the shortest pulse duration showed a presence of the $\theta\cong(0.15-0.5)^{\circ}$ pulse front tilt (Fig. S1(c)) for $\gamma\cong\mp2 \text{fs}^{-2}$ (i.e., pulse duration was changed by a factor 2.2 times as compared to the shortest pulse setting); noteworthy the sign of the pulse front tilt was always the same. The spatial chirp can be considered negligible at the compressor position corresponding to the shortest pulse (close to the bandwidth limited duration).

For the focusing of the 1 µm wavelength laser pulses with a commercial objective lens optimised for the visible spectral range, it is expected to increase the pulse front tilt. Even for exaggerated $\theta=1^{\circ}$the difference between the left- and right-side (see the top-view image in the inset of Fig. S1(c)) incidence correspond to only $d\tan(1^{\circ})\cong22 \text{nm}$ for the *d* = 2.5 µm-diameter focal spot or ~0.2 fs in time inside the glass. Even though these values appear small, they can still play an important role at ~ 1 TW/cm^2^ peak intensities in the electron generation rate since plasma density can change by a factor of two (or more) within of an optical cycle of ~ 3.5 fs^2^. The electron-ion relaxation time is even shorter ~ 1.1 fs^3^ at the glass breakdown conditions and the “snow plow” effect pushing electrons by the tilted pulse front can influence the formation of self-trapped excitons and defect formation at later times (~ µs).

The first observation of the “snow plow” effect^4^ was analysed considering much higher intensities and several degrees of the front tilt at which there was the difference in structural changes of glass along two opposite directions of a scan.

| 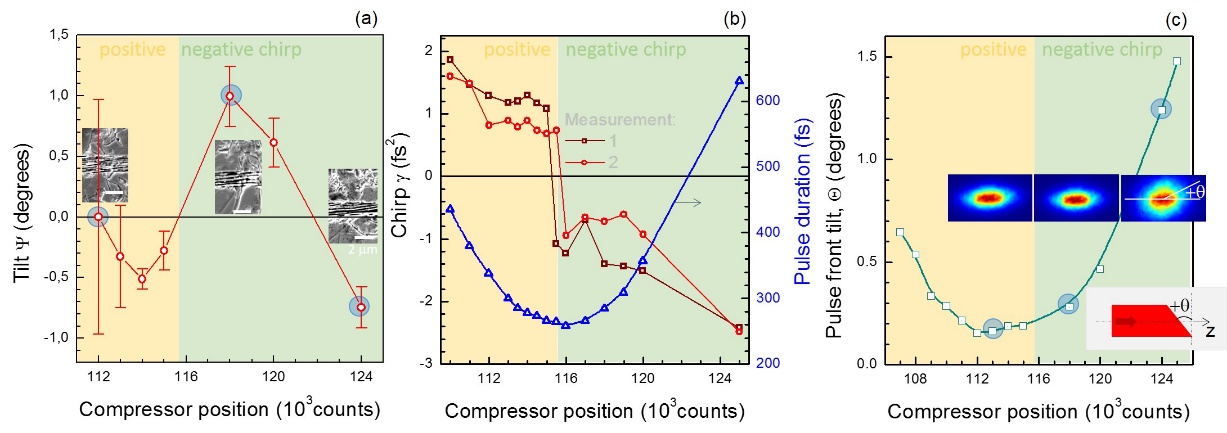 |
| --- |

**Figure S1**. **Spatial and temporal chirp influence to the nanogratings tilt angle.** (a) Tilt angle $\Psi$ vs. compressor position (a measure for a temporal chirp,$\gamma$). Insets show corresponding SEM images of nanogratings at three different compressor positions. (b) The chirp, $\gamma\text{[fs}^{-2}\text{]}$and pulse duration,$\tau_{p}$, dependence on the compressor setting. Two scans of compressor stage in positive and negative directions were carried out. (c) Spatial chirp, the pulse front tilt vs. the compressor position. Insets show corresponding second harmonic intensity distribution profile measured by single shot autocorrelation; top inset shows schematically the spatial chirp (pulse front tilt) along the propagation (z-axis). Laser pulses were$\lambda=1030 \text{nm}$, $\tau_{p}=260 \text{fs}$ (Pharos).

**References**

1. Juodkazis, S., Nishi, Y., Misawa, H., Mizeikis, V., Schecker, O., et al. Optical transmission and laser structuring of silicon membranes. Opt. Express **17**(17), 15308-15317 (2009).

2. Malinauskas, M., Žukauskas, A., Bičkauskaitė, G., Gadonas, R. & Juodkazis, S. Mechanisms of three-dimensional structuring of photo-polymers by tightly focused femtosecond laser pulses. Opt. Express **18**(10), 10209-10221 (2010).

3. Hayasaki, Y., Iwata, K., Hasegawa, S., Takita, A. & Juodkazis, S. Time-resolved axial-view of the dielectric breakdown under tight focusing in glass. Opt. Mater. Express **1**, 1399-1408 (2011).

4. Kazansky, P. G., Yang, W., Bricchi, E., Bovatsek, J., Arai, A., et al. “quill” writing with ultrashort light pulses in transparent materials. Appl. Phys. Lett. **90**, 151120 (2007).
